# Long COVID symptoms after 8-month recovery: persistent static lung hyperinflation associated with small airway dysfunction

**DOI:** 10.1186/s12931-024-02830-1

**Published:** 2024-05-15

**Authors:** Po-Chun Lo, Jia-Yih Feng, Yi-Han Hsiao, Kang-Cheng Su, Kun-Ta Chou, Yuh-Min Chen, Hsin-Kuo Ko, Diahn-Warng Perng

**Affiliations:** 1 Department of Chest Medicine, Taipei Veterans General Hospital, No. 201, Sec. 2, Shih-Pai Road, Taipei 112, Taipei, 11217 Taiwan, ROC; 2 College of Medicine, National Yang Ming Chiao Tung University, Taipei, 11221 Taiwan, ROC; 3 Department of Internal Medicine, Taoyuan General Hospital, Ministry of Health and Welfare, Taoyuan, Taiwan; 4 Institute of Emergency and Critical Care Medicine, College of Medicine, National Yang Ming Chiao Tung University, Taipei, 11221 Taiwan, ROC

**Keywords:** COVID-19, Post-acute sequelae of COVID-19, Small airway dysfunction, Static lung hyperinflation

## Abstract

**Background:**

Limited research has investigated the relationship between small airway dysfunction (SAD) and static lung hyperinflation (SLH) in patients with post-acute sequelae of COVID-19 (PASC) especially dyspnea and fatigue.

**Methods:**

64 patients with PASC were enrolled between July 2020 and December 2022 in a prospective observational cohort. Pulmonary function tests, impulse oscillometry (IOS), and symptom questionnaires were performed two, five and eight months after acute infection. Multivariable logistic regression models were used to test the association between SLH and patient-reported outcomes.

**Results:**

SLH prevalence was 53.1% (34/64), irrespective of COVID-19 severity. IOS parameters and circulating CD4/CD8 T-cell ratio were significantly correlated with residual volume to total lung capacity ratio (RV/TLC). Serum CD8 + T cell count was negatively correlated with forced expiratory volume in the first second (FEV_1_) and forced vital capacity (FVC) with statistical significance. Of the patients who had SLH at baseline, 57% continued to have persistent SLH after eight months of recovery, with these patients tending to be older and having dyspnea and fatigue. Post-COVID dyspnea was significantly associated with SLH and IOS parameters R5-R20, and AX with adjusted odds ratios 12.4, 12.8 and 7.6 respectively. SLH was also significantly associated with fatigue.

**Conclusion:**

SAD and a decreased serum CD4/CD8 ratio were associated with SLH in patients with PASC. SLH may persist after recovery from infection in a substantial proportion of patients. SAD and dysregulated T-cell immune response correlated with SLH may contribute to the development of dyspnea and fatigue in patients with PASC.

**Supplementary Information:**

The online version contains supplementary material available at 10.1186/s12931-024-02830-1.

## Background

Post-acute sequelae of COVID-19 (PASC), also known as long COVID, is a syndrome that affects multiple organs after a SARS-CoV-2 infection. The World Health Organization defines PASC as involving symptoms that are present three months after SARS-CoV-2 infection, that have a duration of at least two months, and that cannot be explained by an alternative diagnosis [1]. Subramanian et al. analyzed a UK primary care database and reported that 62 symptoms were significantly associated with SARS-CoV-2 infection at 12 weeks in non-hospitalized adults [2]. On the basis of conservative models, the estimated global proportion of PASC was approximately 6.2–12.7% [3, 4]. Notably, dyspnea, fatigue, and neurocognitive impairment are the most common symptoms reported in patients with PASC [5–7], and several systematic reviews have indicated that approximately 18–41% of the patients had breathlessness after COVID-19 [8, 9]. Even patients with mild COVID-19 had an increased risk of persistent dyspnea and weakness at one-year follow-up [10]. Post-COVID-19 breathlessness significantly impairs recovery and reduces working capacity [6].

The mechanisms underlying post-COVID-19 breathlessness are not fully understood [8, 11]. Post-COVID-19 breathlessness has been correlated with pulmonary function abnormalities [12], including restrictive ventilatory impairment and decreased lung diffusing capacity [5, 12, 13] in patients with SARS-CoV-2 infection 4–6 months after discharge from the hospital [14]. Cho et al. also identified small airway dysfunction (SAD), air-trapping, and static lung hyperinflation (SLH) [15] in patients with PASC; moreover, 88.1% of patients with PASC had abnormal impulse oscillometry (IOS) parameters [16]. IOS uses an oscillation technique in an effort-independent manner to measure airway resistance and reactance. We previously reported that respiratory reactance at 5 Hz (X5) can predict SLH in severe asthma with high sensitivity and specificity [17]. Clinically, dyspnea in patients with PASC is associated with abnormal lung function. However, whether SAD and SLH are associated with respiratory symptoms in PASC remains unclear.

We hypothesized that SARS-CoV-2 infection leads to SAD and contributes to SLH, which is associated with respiratory symptoms in patients with PASC. In this prospective observational cohort study, we determined the interrelationship between small airway function, SLH, and respiratory symptoms in patients with PASC eight months after SARS-CoV-2 infection.

## Methods

### Study design and participant selection

This single-center, prospective, observational cohort study was approved by the Institutional Review Board of Taipei Veterans General Hospital (VGHTPE-IRB No. 2020-07-011CC). We enrolled consenting adults aged over 20 years with COVID-19 confirmed through polymerase chain reaction who remained symptomatic for two months between July 2020 and December 2022. Eligible participants were referred to the post-acute COVID-19 clinic at Taipei Veterans General Hospital. We excluded participants with a history of chronic lung disease, including chronic obstructive pulmonary disease, asthma, bronchiectasis, lung cancer, pulmonary fibrosis, pulmonary tuberculosis, or any neuromuscular or spinal disease that affected lung function and small airway function.

We collected demographic information and conducted the following examinations at the first (two months after diagnosis), second (five months after diagnosis) and third (eight months after diagnosis) follow-up visits (visits 1, 2, and 3 respectively): (1) symptom assessment and administration of St. George’s Respiratory Questionnaire (SGRQ); (2) administration of spirometry, lung volume, diffusing capacity of the lung for carbon monoxide (DLCO), 6-min walk test (6MWT), and IOS; and (3) analysis of serum and plasma samples for CD4 + and CD8 + T-cell counts. The data collected at visit 1 were considered as baseline data for analysis.

### Symptom assessment

At each visit, the participants with PASC were asked to complete a structured questionnaire, which collected data related to the following symptoms: fatigue, cough, dyspnea, phlegm, anosmia, dysgeusia, sleep disturbance, alopecia, myalgia, upper respiratory symptoms, nausea, headache, diarrhea, lightheadedness, palpitations, chest pain, brain fog, and skin rash.

### Pulmonary function tests

Pulmonary function tests were performed according to the manufacturer’s recommendations and the American Thoracic Society and the European Respiratory Society (ERS) guidelines [18] using the Spiro Medics system 2130 (SensorMedics, Anaheim, CA, USA). SLH was defined as a residual volume to total lung capacity ratio (RV/TLC) ≥ 40% [19]. Impulse oscillometry (Jaeger MS-IOS, Germany) was performed before spirometry, and measurements were performed in triplicate according to the ERS technical standards [20]. IOS have better sensitivity than forced expiratory flow between 25% and 75% (FEF25%-75%) to detect SAD [21]. The cutoffs for SAD were the difference between resistance at 5 and 20 Hz (R5-R20) greater than 0.07 kPa/(L/s), the area under the reactance curve between 5 Hz and the resonant frequency (AX) greater than 0.44 kPa/L, X5 less than − 0.12 kPa/(L/s), or resonant frequency (Fres) greater than 14.14 Hz [21]. Exercise-induced hypoxemia (EIH) was defined as a drop of 4% in SpO2 during exercise [22].

### Statistics

Continuous variables are reported as median and interquartile range (IQR) or mean with standard error measures. Categorical variables are reported as counts and percentages. Data were tested for normality using the Shapiro-Wilk before analysis. We compared the data between the ambulatory, hospitalized, and intensive care unit (ICU) groups by using the Kruskal–Wallis test for nonnormally distributed variables and the chi-square test for categorical variables. Comparisons between the SLH and non-SLH groups were performed using the Mann–Whitney U test for continuous variables and Fisher’s exact test for categorical variables. Variables were compared between visits 1, 2, and 3 by using paired-samples t-tests and McNemar’s test. Spearman’s rank correlation test was used to determine associations between the RV/TLC ratio and clinical variables. Univariable and multivariable logistic regression analyses were used to test associations between symptoms, the presence of SLH, and clinical variables. Clinically meaningful variables and those with *p* < 0.15 in the univariable logistic regression were subsequently analyzed in the multivariable model. R5-R20, AX, Fres, and X5 were highly correlated and were found to have multicollinearity [23]. Therefore, a separate multivariate logistic regression model was used for each IOS parameter for predicting long COVID symptoms, and these were subsequently adjusted for age, sex, smoking, COVID-19 severity, and body mass index (BMI). Seventeen participants missed their visits during the follow-up period, and we applied listwise deletion in the logistic regression model for the association between SLH and long COVID symptoms at visit 3. *P* values < 0.05 were taken as statistically significant. All data were analyzed using SPSS for Windows (version 25.0, IBM, Armonk, NY, USA) and plotted using OriginPro 2022 (OriginLab Corporation, version SR1, Northampton, MA, USA) and GraphPad Prism (version 9; La Jolla, CA, USA).

## Results

### Baseline characteristics of the study population

**Fig. 1:**
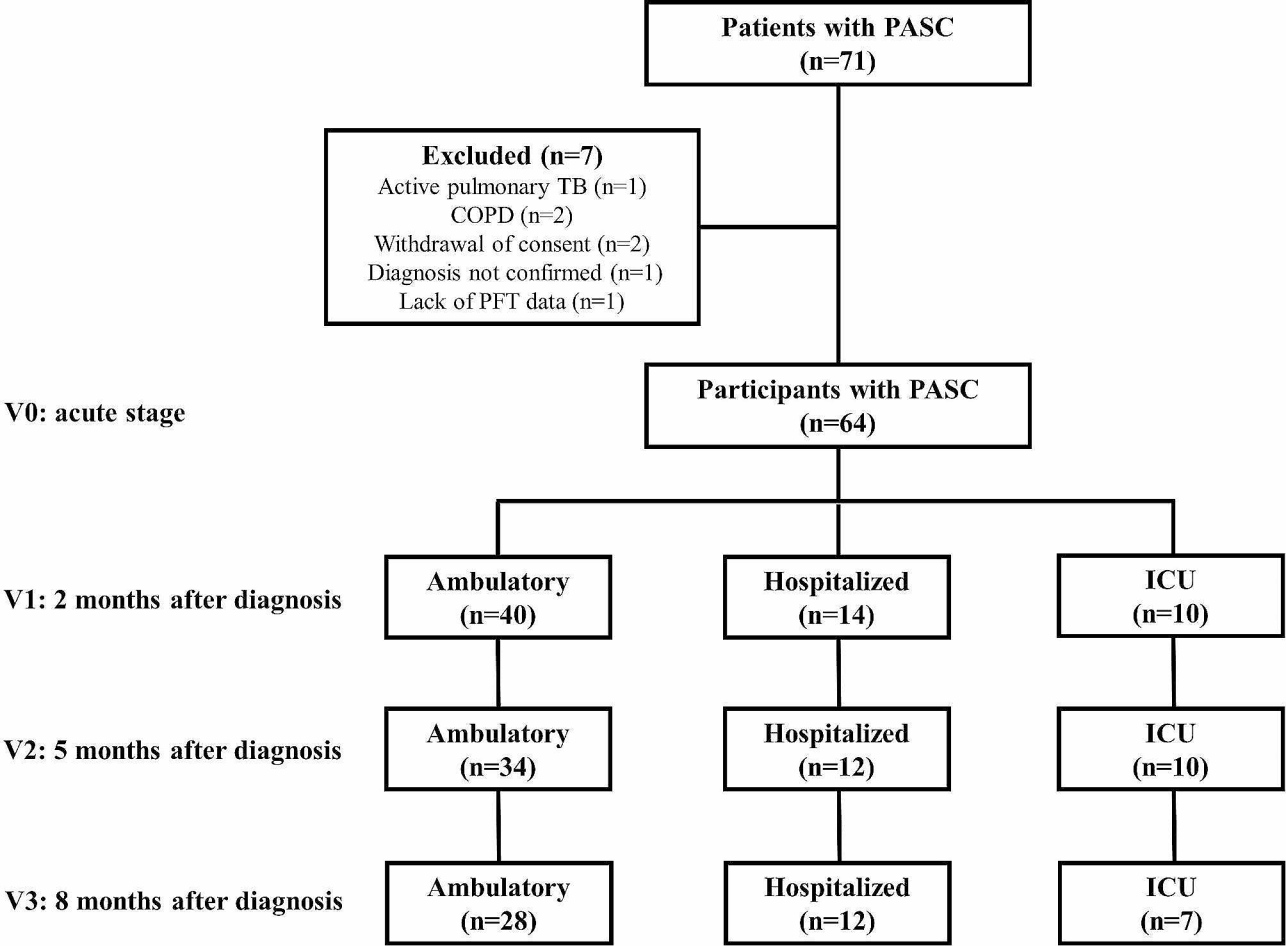
Study flowchart A total of 64 participants with PASC were enrolled in this prospective cohort study. We stratified the participants based on their acute phase severity into ambulatory (*n* = 40), hospitalized (*n* = 14), and ICU (*n* = 10) groups. Participants were followed up at visits 1 (2 months after diagnosis), 2 (5 months after diagnosis) and 3 (8 months after diagnosis). 17 participants missed their visits during the follow-up period. COPD = chronic obstructive pulmonary disease, ICU = intensive care unit, PASC = post-acute sequelae of COVID-19, PFT = pulmonary function test, TB = tuberculosis

We included 64 participants with PASC (mean age, 56.7 years; 28 men) who were ambulatory (*n* = 40), hospitalized (*n* = 14), or admitted to the ICU (*n* = 10; Fig. 1). Their demographic and baseline clinical characteristics are presented in Table 1. The mean age and median BMI were not significantly different between the groups. SLH prevalence was 53.1% (34/64), irrespective of the COVID-19 severity. The PASC patients exhibited 10.9% (7/64) with FEV1% predicted < 80%, 87.5% (56/64) with DLCO%pred < 80% and no airway obstruction (FEV1/FVC < 0.7) (Table 2). The percentage of abnormality of IOS parameters meeting our SAD cutoff were 43.8% in R5-R20, 56.3% in AX, 54.7% in X5, and 57.8% in Fres (Table 2). The clinical symptoms of the patients with PASC are summarized in Supplementary Table A, with the most commonly reported being fatigue (57.8%), followed by brain fog (50.0%) and respiratory symptoms, including mucus secretion (46.9%), cough (43.8%), and dyspnea (43.8%).

**Table 1:** Clinicodemographic characteristics of participants with PASC at visit 1

Characteristic	PASC	Ambulatory Group	Hospitalized Group	ICU Group	*p* value†
Numbers (%)	64 (100)	40 (62.5)	14 (21.9)	10 (15.6)	
Age, yr	56.7 ± 13.1	56.7 ± 13.8	54.57 ± 10.5	60.0 ± 13.8	0.643
Male (%)	28 (43.8)	13 (32.5)	8 (57.1)	7 (70)	0.053
BMI, kg/m2	25.4 (22.5–29.5)	24.4 (22.3–30.0)	26.5 (25.4–32.1)	24.4 (23.7–27.7)	0.196
Smoking history					**0.009**
Never smoker (%)	52 (81)	37 (92.5)	8 (57.1)	7 (70)	
Former smoker (%)	2 (3)	0 (0)	2 (14.3)	0 (0)	
Current smoker (%)	10 (16)	3 (7.5)	4 (28.6)	3 (30)	
Comorbidity					
Cancer (%)	5 (7)	3 (7)	1 (7)	1 (10)	0.961
CKD (%)	2 (3)	2 (5)	0 (0)	0 (0)	0.544
CAD (%)	4 (6)	1 (3)	2 (14)	1 (10)	0.259
Heart failure (%)	5 (7)	2 (5)	0 (0)	3 (30)	0.291
Hypertension (%)	21 (33)	11 (28)	6 (43)	4 (40)	0.293
Diabetes type II (%)	14 (22)	5 (13)	6 (43)	3 (30)	0.051
Chronic hepatitis B (%)	4 (6)	3 (7)	0 (0)	1 (10)	0.474
SLE (%)	2 (3)	2 (5)	0 (0)	0 (0)	0.544
Presence of SLH (%)	34 (53.1)	23 (57.5)	5 (35.7)	6 (60)	0.333
Questionnaires					
SGRQ	11.34 (3.39–25.53)	13.67 (4.06–24.09)	5.61 (0.94–11.40)	22.76 (2.33–42.24)	0.146
Fatigue (%)	37 (57.8)	25 (62.5)	5 (35.7)	7 (70)	0.152
Dyspnea (%)	28 (43.8)	17 (42.5)	5 (35.7)	6 (60)	0.481
Brain fog (%)	32 (50)	24 (60)	4 (28.5)	4 (40)	0.102
Cough (%)	28 (43.8)	16 (40)	7 (50)	7 (70)	0.737
Mucus secretion (%)	30 (46.9)	17 (42.5)	7 (50)	6 (60)	0.590

BMI: body mass index, CAD: coronary artery disease, CKD: chronic kidney disease, ICU: intensive care unit, PASC: post-acute sequelae of COVID-19, SGRQ: St George’s Respiratory Questionnaire, SLE: systemic lupus erythematosus, SLH: static lung hyperinflation

† Subgroup comparison

Bold text indicates that the *p* value is significant

**Table 2:** Pulmonary function, IOS parameters and serum T cell count of patients with PASC at visit 1

Characteristic	PASC
Numbers	64
Pulmonary Function	
FVC, L	2.84 ± 0.85
FVC % pred.	89.3 ± 14.3
FEV1, L	2.40 ± 0.65
FEV1% pred	95.9 ± 12.6
FEV1% pred < 80% (%)	7 (10.9)
FEV1/FVC, %	85.1 ± 6.5
FEV1/FVC < 0.7 (%)	0 (0)
TLC, L	4.42 (3.76–5.30)
TLC, % pred.	96.7 ± 14.2
RV, % pred.	110.0 (93.5–128.0)
RV/TLC, %	40.0 ± 6.5
DLCO, % pred.	63.0 ± 13.7
DLCO % pred. <80% (%)	56 (87.5)
R5-R20 [kPa/(L/s)]	0.07 (0.04–0.12)
R5-R20 > 0.07 (%)	28 (43.8)
AX, kPa/L	0.53 (0.27–0.93)
AX > 0.44 (%)	36 (56.3)
X5, kPa/(L/s)	-0.12 (-0.18- -0.08)
X5 < -0.12 (%)	35 (54.7)
Fres, Hz	14.85 (12.03–17.15)
Fres > 14.14 (%)	37 (57.8)
SAD (%)	42 (65.6)
CD4 + T cell, cell/uL	666.0 (451.5–890.0)
CD8 + T cell, cell/uL	397.0 (294.0-590.0)
CD4/CD8 T cell ratio	1.54 (1.09–2.39)

% pred.: % of predicted value, AX: area under reactance curve between 5 Hz and resonant frequency, DLCO: diffusing capacity of the lung for carbon monoxide, FEV1: forced expiratory volume in 1 s, Fres: resonant frequency, FVC: forced vital capacity, IOS: impulse oscillometry, PASC: post-acute sequelae of COVID-19, R5-R20: difference between resistance at 5 and 20 Hz, RV: residual volume, RV/TLC: residual volume to total lung capacity ratio, SAD: small airway dysfunction, X5: reactance in 5 Hz

The cutoffs for SAD were the difference between resistance at 5 and 20 Hz (R5-R20) greater than 0.07 kPa/(L/s), the area under the reactance curve between 5 Hz and the resonant frequency (AX) greater than 0.44 kPa/L, X5 less than − 0.12 kPa/(L/s), or resonant frequency (Fres) greater than 14.14 Hz

### Clinical features of patients with PASC with and without SLH

The patients with PASC and SLH were older and had lower BMI, and RV/TLC ratio than those without SLH (Table 3). However, FEV1 (% pred), FVC (% pred), total lung capacity (TLC % pred), residual volume (RV % pred), EIH and COVID-19 severity were not significantly different between the groups. Compared to patients without SLH, those with SLH had significantly higher values of the IOS parameters R5-R20, AX, and Fres, lower values of CD4/CD8 T cell ratio and higher prevalence of fatigue and dyspnea. The percentage of SAD was significantly higher in the SLH group compared to the non-SLH group.

**Table 3:** Comparison of characteristics between patients with PASC with and without static lung hyperinflation at visit 1

Characteristic	Non-SLH	SLH	*p*-value
Numbers (%)	30 (46.9)	34 (53.1)	
Age, yr	51.0 ± 10.7	61.8 ± 13.0	**0.002**
Female (%)	13 (43.3)	23 (67.6)	0.077
BMI, kg/m2	27.2 (24.3–32.0)	24.1 (22.4–26.2)	**0.027**
Smoking history			0.293
Never smoker (%)	23 (77)	29 (85)	
Former smoker (%)	5 (17)	5 (15)	
Current smoker (%)	2 (7)	0 (0)	
COVID-19 severity			0.333
Ambulatory (%)	17 (56.7)	23 (67.6)	
Hospitalized (%)	9 (30.0)	5 (14.7)	
ICU (%)	4 (13.3)	6 (17.6)	
Pulmonary Function			
FVC, % pred.	92.9 ± 14.7	86.0 ± 13.3	0.07
FEV1, % pred	97.2 ± 12.8	94.8 ± 12.6	0.423
FEV1/FVC %	83.9 ± 5.6	86.2 ± 7.2	0.159
FEF25%-75%, % pred	89.8 ± 23.2	96.6 ± 33.1	0.472
TLC, % pred.	97.1 ± 13.9	96.3 ± 14.6	0.767
RV, % pred.	106.0 ± 19.6	117.3 ± 25.4	0.089
RV/TLC %	34.6 ± 3.7	44.7 ± 4.3	**< 0.001**
DLCO, % pred.	65.4 ± 13.2	60.7 ± 14.0	0.114
R5-R20 [kPa/(L/s)]	0.07 (0.03–0.10)	0.09 (0.06–0.16)	**0.039**
R5-R20 > 0.07 (%)	10 (33.3)	18 (52.9)	0.136
AX (kPa/L)	0.36 (0.15–0.75)	0.60 (0.32–1.17)	**0.030**
AX > 0.44 (%)	13 (43.3)	23 (67.6)	0.077
X5 [kPa/(L/s)]	-0.10 (-0.18- -0.07)	-0.13 (-0.18- -0.10)	0.140
X5 < -0.12 (%)	13 (43.3)	22 (64.7)	0.131
Fres (Hz)	14.34 (9.42–15.66)	15.35 (13.25–17.88)	**0.030**
Fres > 14.14 (%)	15 (50.0)	22 (64.7)	0.312
SAD (%)	15 (50.0)	27 (79.4)	**0.018**
6MWD, meter	402 (363–446)	407 (340–447)	0.989
EIH (%)	4 (13.3)	7 (20.6)	0.520
SGRQ	7.68 (2.98–22.71)	13.67 (3.86–25.58)	0.459
Fatigue (%)	11 (36.7)	26 (76.5)	**0.002**
Dyspnea (%)	7 (23.3)	21 (61.8)	**0.003**
Brain fog (%)	12 (40.0)	20 (58.8)	0.210
Cough (%)	14 (46.7)	14 (41.2)	0.801
Mucus secretion (%)	12 (40.0)	18 (53.0)	0.327
CD4^+^ T cell, cell/uL	717 (557–1047)	591 (387–845)	0.051
CD8^+^ T cell, cell/uL	369 (250–522)	430 (337–522)	0.239
CD4/CD8 T cell ratio	2.20 (1.31–2.87)	1.19 (0.88–1.95)	**0.001**
Blood eosinophil, cell/uL	142 (59–221)	106 (68–161)	0.282

% pred.: % of predicted value, 6MWD: 6-min walking distance, AX: area under reactance curve between 5 Hz and resonant frequency, BMI: body mass index, DLCO: diffusing capacity of the lung for carbon monoxide, EIH: exercise-induced hypoxemia, FEV1: forced expiratory volume in 1 s; FEF25%-75%: Forced expiratory flow at 25–75% of FVC; Fres: resonant frequency, FVC: forced vital capacity, ICU: intensive care unit, PASC: post-acute sequelae of COVID-19, R5-R20: difference between resistance at 5 and 20 Hz, RV: residual volume, RV/TLC: residual volume to total lung capacity ratio, SGRQ: St George’s Respiratory Questionnaire, SAD: small airway dysfunction, SLH: static lung hyperinflation. X5: reactance in 5 Hz.

Bold text indicates that the *p* value is significant.

The cutoffs for SAD were the difference between resistance at 5 and 20 Hz (R5-R20) greater than 0.07 kPa/(L/s), the area under the reactance curve between 5 Hz and the resonant frequency (AX) greater than 0.44 kPa/L, X5 less than − 0.12 kPa/(L/s), or resonant frequency (Fres) greater than 14.14 Hz

### Correlation between RV/TLC, IOS parameters, and serum T-cell counts

**Fig. 2:**
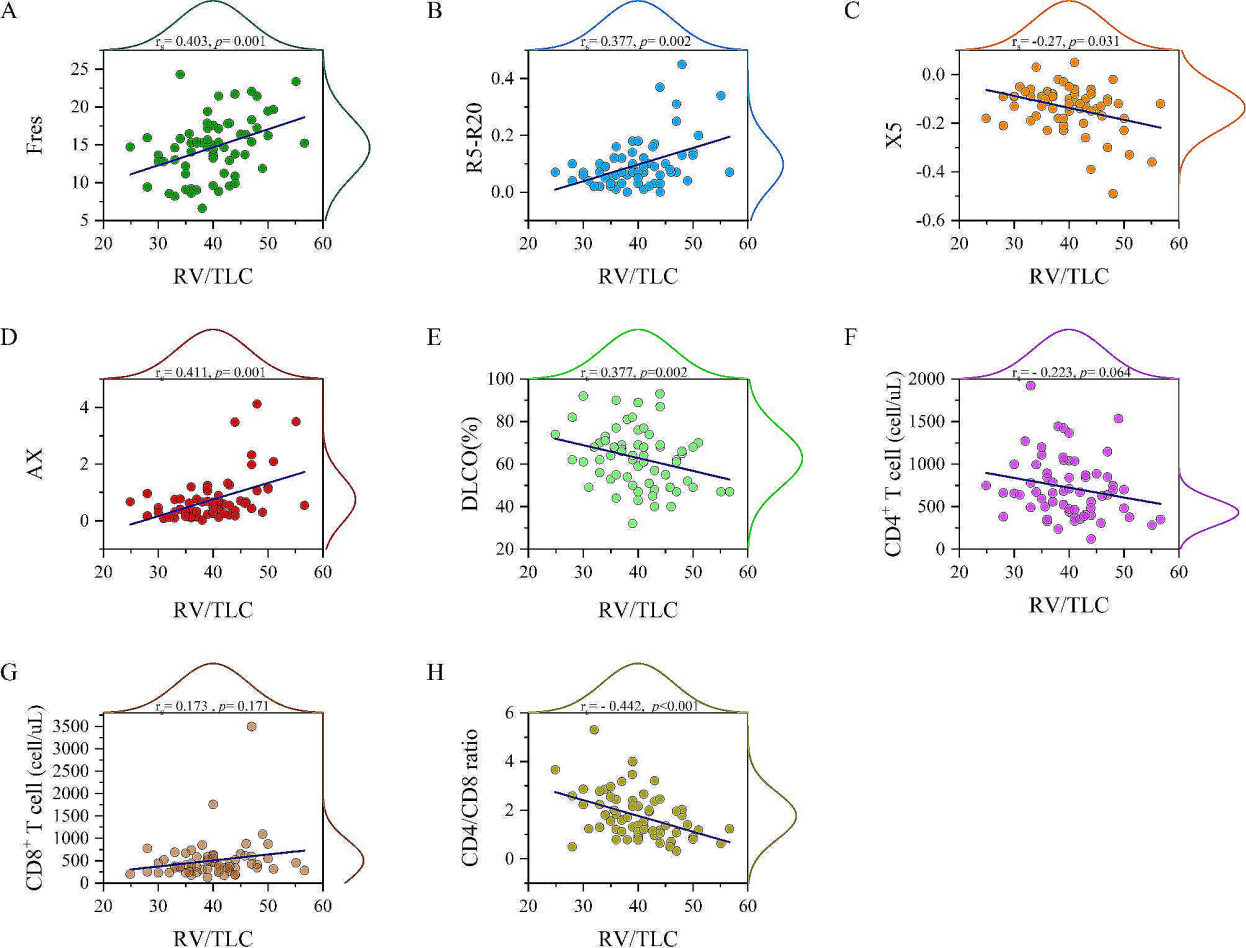
Correlations between RV/TLC, impulse oscillometry parameters, and serum T-cell count Correlations are presented using a scatter plot and marginal histogram. (**A**) Resonant frequency (Fres), (**B**) difference between resistance at 5 Hz and 20 Hz (R5-R20), (**C**) reactance at 5 Hz (X5), (**D**) area under the reactance curve between 5 Hz and resonant frequency (AX), (**E**) DLCO%, (**F**) CD4 + T-cell count, (**G**) CD8 + T-cell count, and (**H**) CD4/CD8 T-cell count. DLCO = diffusing capacity of the lung for carbon monoxide, RV = residual volume, TLC = total lung capacity

All IOS parameters (R5-R20, X5, AX, and Fres) and DLCO were significantly correlated with RV/TLC (Fig. 2). Serum T-cell counts and CD4/CD8 ratios are presented in Table 2. The decrease in CD4 + and increase in CD8 + T-cell counts reduced the CD4/CD8 ratio in the SLH group, which was significantly correlated with RV/TLC (Fig. 2). Furthermore, CD8 + T-cell counts, but not CD4 + T-cell counts, were negatively correlated with FEV1 (% pred, *p* = 0.006) and FVC (% pred, *p* = 0.011).

### Dynamic changes of lung function and long COVID symptoms

**Fig. 3:**
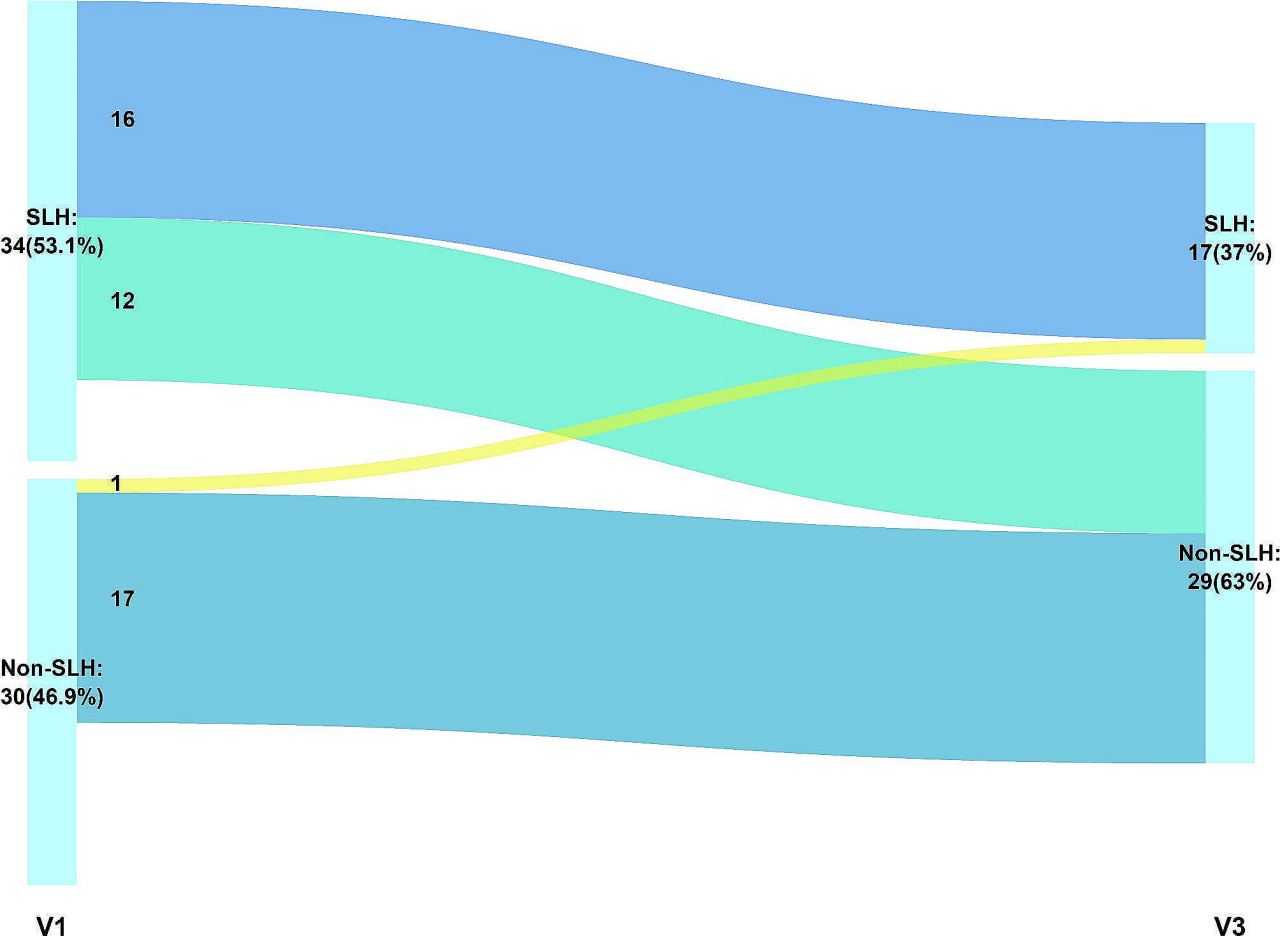
Sankey diagrams for SLH transition Flows are color-coded as the following state: light blue flows have persistent SLH at the visit 3, light green flows have a recovery to non-SLH at the visit 3, yellow flows revert to SLH at the visit 3, and blue flows have non-SLH at the visit 3. The numbers in the flow are numbers of participants. SAD = small airway dysfunction, SLH = static lung hyperinflation, V = visit

Six months (V3) after visit 1, the lung function parameters of FEV1, FVC, TLC, RV/TLC, DLCO % pred and SGRQ scores significantly improved (Table 4). However, FEF25%-75%, IOS parameters (R5-R20, X5, AX, and Fres), percentage of abnormality in IOS parameters and the prevalence of dyspnea, cough and mucin production did not significantly change between visits. However, the prevalence of SLH and fatigue showed a significant decrease between PASC and 6 months after PASC (53.1% vs. 37.0%, *p* = 0.003; 57.8% vs. 40.4%, *p* = 0.019, respectively). To explore the transition of SLH in patients with PASC between visits, a Sankey diagram is presented in Fig. 3, illustrating 37% of the patients (17/46) were SLH. Among the patients with SLH, 57% (16/28) had persistence of SLH, and 43% (12/28) experienced recovery. 6% of patients without SLH (1/18) developed lung hyperinflation between visits. Patients with persistent SLH were characterized by older age, a higher prevalence of dyspnea and fatigue, R5-R20 > 0.07 kPa/(L/S), and a lower serum B cell count at visit 1 (supplementary Table B).

**Table 4:** Changes in pulmonary function and symptoms between visits 1, 2 and 3

Characteristic	V1	V2	V3	*p*-value^†^	*p*-value^#^
Numbers	64	56	47		
Presence of SLH (%)	34 (53.1)	25 (44.6)	17(37.0)^※^	0.180	**0.003**
Pulmonary Function					
FVC, % pred.	89.3 ± 14.3	93.2 ± 11.57	96.0 ± 11.6	**0.003**	**< 0.001**
FEV1, % pred	95.9 ± 12.6	99.0 ± 10.5	101.5 ± 11.0	**0.039**	**< 0.001**
FEV1 % pred < 80, (%)	7 (10.9)	3 (5.4)	2 (4.3)	0.5	0.25
FEF25%-75%, % pred	93.4 ± 28.9	89.9 ± 24.8	89.4 ± 24.5	0.151	0.055
FEF25%-75% % pred < 65, (%)	8 (12.5)	11 (19.6)	7 (14.9)	0.289	0.625
TLC, % pred.	96.7 ± 14.2	99.8 ± 12.3	101.8 ± 11.2^※^	**0.014**	**0.004**
RV/TLC %	40.0 ± 6.5	39.1 ± 6.6	38.7 ± 7.7^※^	0.246	**0.031**
DLCO, % pred.	63.0 ± 13.7	67.3 ± 13.2	68.0 ± 12.8	**0.005**	**0.009**
DLCO % pred. <80, (%)	56 (87.5)	44 (78.6)	36 (78.3)	0.289	0.219
R5-R20 [kPa/(L/s)]	0.07 (0.04–0.12)	0.08 (0.04–0.11)	0.08 (0.04–0.11)	0.116	0.657
R5-R20 > 0.07 (%)	28 (43.8)	28 (52.8)	25 (53.2)	0.238	0.143
AX (kPa/L)	0.53 (0.27–0.93)	0.50 (0.24–0.76)	0.48 (0.21–0.77)	0.617	0.594
AX > 0.44 (%)	36 (56.3)	31 (56.4)	25 (53.2)	0.791	1.000
X5 [kPa/(L/s)]	-0.12 (-0.18- -0.08)	-0.13 (-0.16- -0.09)	-0.12 (-0.16- -0.09)	0.275	0.673
X5 < -0.12 (%)	35 (54.7)	30 (54.5)	21 (44.7)	1.000	0.302
Fres (Hz)	14.85 (12.03–17.15)	14.31 (11.44–15.69)	14.44 (12.05–15.95)	0.109	0.334
Fres > 14.14 (%)	37 (57.8)	26 (47.3)	26 (55.3)	0.454	1.000
SAD (%)	42 (65.6)	35 (63.6)	33 (70.2)	1.000	0.754
Questionnaires					
SGRQ	11.34 (3.39–25.53)	5.94 (0.00-16.10)	1.71 (0.00-11.47)	**0.007**	**< 0.001**
Symptoms					
Fatigue (%)	37(57.8)	31 (55.4)	19 (40.4)	0.180	**0.019**
Dyspnea (%)	28(43.8)	20 (35.7)	17 (36.2)	0.302	0.481
Brain fog (%)	32 (50.0)	21 (37.5)	17 (36.2)	0.227	0.125
Cough (%)	28 (43.8)	16 (28.6)	10 (21.3)	0.077	0.057
Mucus secretion (%)	30 (46.9)	29 (52.7)	19 (40.4)	0.481	1.000

† Comparison of visit 1 and 2

# Comparison of visit1 and 3

※ 46 patients had analyzed

% pred.: % of predicted value, AX: area under reactance curve between 5 Hz and resonant frequency, DLCO: diffusing capacity of the lung for carbon monoxide, FEV1: forced expiratory volume in 1 s; FEF25–75%: Forced expiratory flow at 25–75% of FVC, Fres: resonant frequency, FVC: forced vital capacity, R5-R20: difference between resistance at 5 and 20 Hz, RV: residual volume, RV/TLC: residual volume to total lung capacity ratio, SGRQ: St George’s Respiratory Questionnaire, SAD: small airway dysfunction, SLH: static lung hyperinflation, V1: visit one, V2: visit two, V3: visit three, X5: reactance in 5 Hz. Bold text indicates that the *p* value is significant

The cutoffs for SAD were the difference between resistance at 5 and 20 Hz (R5-R20) greater than 0.07 kPa/(L/s), the area under the reactance curve between 5 Hz and the resonant frequency (AX) greater than 0.44 kPa/L, X5 less than − 0.12 kPa/(L/s), or resonant frequency (Fres) greater than 14.14 Hz

### Relationship between clinical variables, dyspnea, and fatigue at visits 2 and 3

**Fig. 4:**
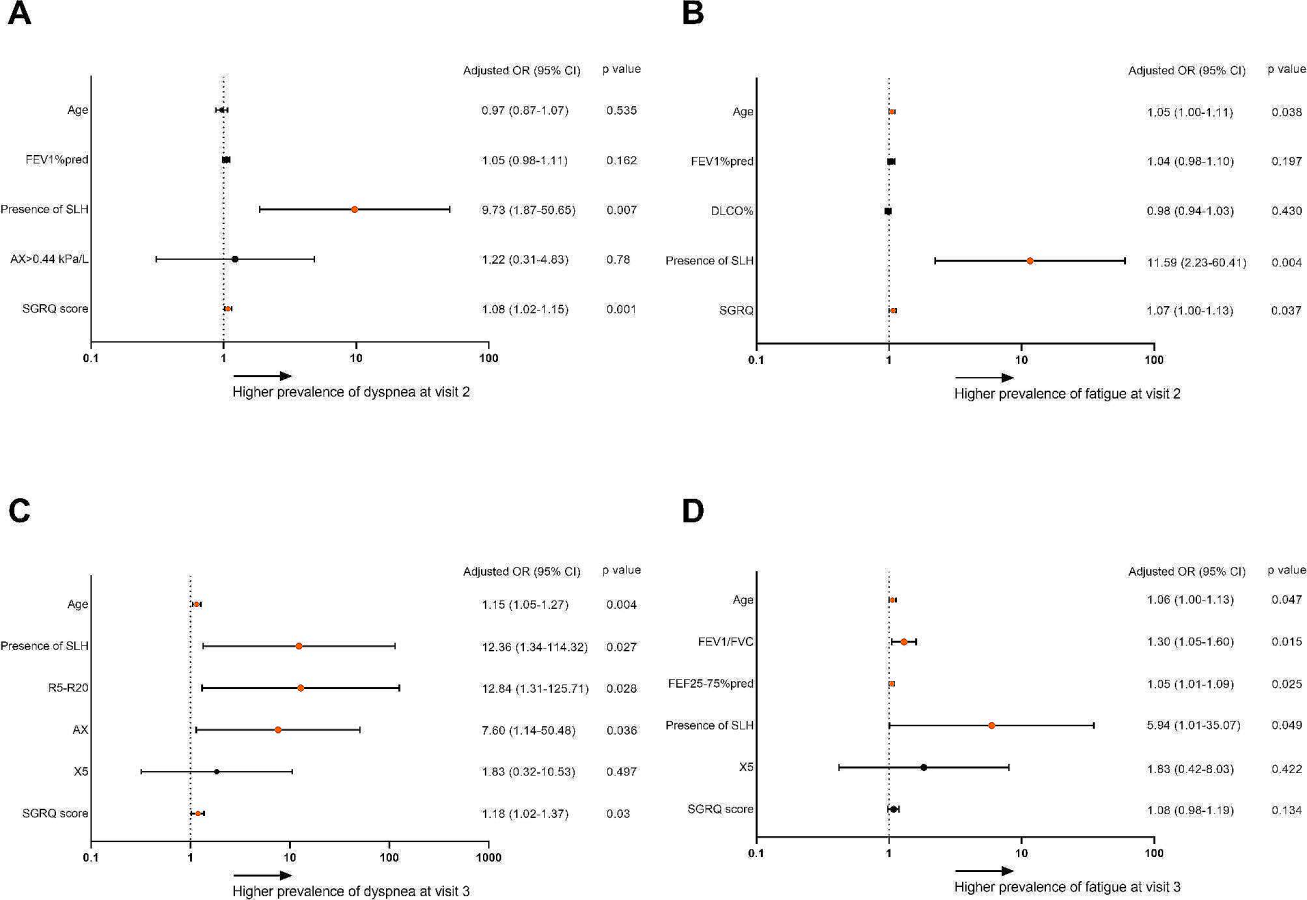
SLH predicts dyspnea and fatigue 5 and 8 months after recovery The logistic regression analysis at 5 and 8 months post recovery is displayed in a forest plot with adjusted odds ratios (aORs) and 95% confidence intervals (CIs) for (**A**) dyspnea visit 2, (**C**) dyspnea at visit 3, (**B**) fatigue at visit 2, and (**D**) fatigue at visit 3. The cutoffs for R5-R20 and AX was 0.07 kPa/(L/s) and 0.44 kPa/L, respectively. Orange labels indicate the significant predictors of symptoms based on the 95% CI of the regression model. AX = area under reactance curve between 5 Hz and resonant frequency, DLCO = diffusing capacity of the lung for carbon monoxide, FEF25%-75% = forced expiratory flow at 25% to 75% of FVC, FEV1 = forced expiratory volume in 1 s, FVC = forced vital capacity, SGRQ = St George’s Respiratory Questionnaire, SLH = static lung hyperinflation, V = visit, X5 = reactance at 5 Hz

The results of the multivariable logistic regression for associations between clinical variables, dyspnea, and fatigue at visits 2 and 3 are presented in Fig. 4. SLH was the most crucial factor for dyspnea development at both visits (adjusted odds ratio [aOR]: 9.73; 95% confidence interval [CI]: 1.87–50.65; *p* = 0.007 at visit 2 and aOR: 12.36; 95% CI: 1.34-114.32; *p* = 0.027 at visit 3) (Fig. 4A, C) and fatigue (aOR: 11.59; 95% CI: 2.23–60.41; *p* = 0.004 at visit 2 and aOR: 5.94; 95% CI: 1.01–35.07; *p* = 0.049 at visit 3) (Fig. 4B, D).

At visit 3, both R5-R20 greater than 0.07 kPa/(L/s) and AX greater than 0.44 kPa/L showed significant associations with dyspnea after adjusting for age, sex, severity of infection, history of smoking, and BMI (aOR for R5-R20: 12.84, 95% CI: 1.31-125.71, *p* = 0.028; aOR for AX: 7.60, 95% CI: 1.14–50.48, *p* = 0.036) (Fig. 4A, C). Furthermore, older age was also significantly associated with dyspnea, but lung function parameters or EIH did not predict the development of dyspnea. Detailed analysis of clinical variables associated with dyspnea and fatigue using logistic regression is presented in Supplementary Tables C1–C4. 22% (14/64) patients received bronchodilator treatment during the cohort study. We categorized PASC patients into those with and without the usage of bronchodilators and compared pulmonary function, IOS parameters, and symptoms between visits. Improvement in FEV1%pred and FEV1/FVC was observed in both groups. However, IOS parameters, the percentage of abnormality of IOS parameters, or dyspnea did not improve in either group (see supplement Table D). Univariable logistic regression analyses were further employed to assess the associations between long COVID dyspnea and fatigue, and the usage of bronchodilators. The insignificant result indicated that the usage of bronchodilators does not impact long COVID dyspnea and fatigue (details are provided in supplement Table C1–4).

## Discussion

In this prospective observational study, we demonstrated that long COVID symptoms—dyspnea and fatigue—are independently associated with SLH, which is correlated with small airway resistance and reactance (R5-R20, X5, AX, and Fres) measured using the IOS. In addition, peripheral airways resistance and reactance, measured by R5-R20 and AX, respectively are associated with post-COVID dyspnea. SLH persistence significantly increased the risk of dyspnea and fatigue after COVID-19. The CD4/CD8 ratio was significantly negatively correlated with RV/TLC. The results of our study indicated that SAD and dysregulated T-cell immune response to SARS-CoV-2 infection are independently associated with SLH, significantly contributing to the development of dyspnea and fatigue in patients with PASC.

COVID-19 patients who develop PASC have been reported to have a high prevalence of SAD and air trapping (58%), as confirmed by using CT images [15]. In patients with severe asthma, we previously reported that SAD is associated with SLH and that airway reactance X5 predicts SLH development with a high probability [17]. In patients with chronic obstructive pulmonary disease, reactance, Fres, and AX were better than resistance parameters in stratifying degree of air trapping and were significantly correlated with RV/TLC [24, 25]. IOS is more sensitive than spirometry in detecting SAD in asthma patients without fixed airway obstruction [26]. Additionally, IOS demonstrates greater sensitivity in detecting SAD compared to FEF25%-75% in symptomatic patients with preserved pulmonary function [21]. In this study, 53.1% of patients with PASC had SLH, with a higher prevalence of dyspnea (76.5%), fatigue (61.8%), and SAD (65.6%). We found that SAD, defined by FEF25–75% less than 65%, was observed in 12.5% of our study participants, compared to 65.6% defined by IOS parameters. The result may suggest that IOS may be more sensitive in detecting SAD compared to using FEF25–75% in PASC patients with preserved lung function. Further, SLH was significantly correlated with SAD in patients with PASC. Our results and those of previous studies exploring the relationship between SAD and SLH development in airway and infectious lung diseases provide a new focus on SAD management.

The etiologies and mechanisms underlying the development of PASC remain unknown. Previous studies have reported inconsistent results regarding the association of post-COVID breathlessness with impaired lung function and a reduction of DLCO [8]. In patients with PASC, lung function and DLCO recovered between 6 months and 1 year, but dyspnea persisted [27]. In our study, impaired lung function and reduction of DLCO were not found to be significantly associated with post-COVID dyspnea. We have also demonstrated a significant improvement in lung function and DLCO after an 8-month recovery period. In survivors of COVID-19, quantitative analysis of expiratory chest CT indicated the presence of air-trapping, which was highly correlated with the RV/TLC ratio, and air-trapping persisted for more than 200 days after diagnosis [15]. Here, we observed that IOS parameter, AX, was only moderately correlated with the RV/TLC ratio, and was not significantly correlated with dyspnea at visit 2. Our data indicated that SLH was the major contributor and independent predictor of clinical symptoms, dyspnea, and fatigue in patients with PASC. We also demonstrated a significant association between SAD measured by R5-R20 and AX and post-COVID breathlessness. Despite a significant improvement in lung function, small airways did not show recovery after 8 months. Our findings reveal a possible new mechanism for post-COVID breathlessness. Air-trapping in smokers with preserved lung function was associated with higher all-cause mortality and adverse respiratory outcomes, leading to increased hospital admissions in the future [28]. Patients with SAD were more likely to report respiratory symptoms such as dyspnea, cough, and chronic phlegm, even if they had never smoked [21, 29]. Additionally, SAD in patients with COPD or asthma was found to be associated with more exacerbations, poorer disease control, and increased dyspnea [23, 30, 31]. Some patients recovered from lung hyperinflation between two and eight months after COVID-19 infection, whereas a small proportion of those without SLH developed lung hyperinflation. The reason for the progressive deterioration of SAD leading to SLH in patients with PASC remains unclear. SARS-CoV can damage alveolar pneumocytes and form fibrogranulation tissue in small airways and airspaces [32]. Similar to SARS-CoV, SARS-CoV-2 uses angiotensin-converting enzyme 2 for host cell entry [33], and this protein is more abundant in pulmonary alveoli and small airways than in large airways, as indicated in a study using single-cell analysis data [34, 35] *Mycoplasma pneumoniae*, *Haemophilus influenzae*, influenza virus, and rhinovirus can target small airways and trigger progressive bronchiolitis [36, 37]. Thus, the persistent SAD in patients with PASC may be attributed to a direct SARS-CoV-2 infection over the small airways. Whether SARS-CoV-2 can target the small airways and whether the persistent dysregulated immune reaction leads to SAD warrant further investigation.

A low CD4/CD8 ratio (< 1.5) may reflect immune senescence and may be associated with various disease entities [38]. Zhang et al. reported that the CD4/CD8 ratio was not significantly different between patients with critical and mild COVID-19 [39]. This phenomenon was also observed in the current study. The relative proportions of circulating CD4 + T-cell counts decreased significantly after SARS-CoV-2 infection, while the circulating CD8 + T-cell counts increased [40]. The interaction between alveolar macrophages and T cells may drive persistent alveolar inflammation [41]. Pulmonary DNA vaccination can induce CD8 + T-cell generation to mediate protective antiviral immunity [42]. Further, dysregulated respiratory CD8 + T-cell responses have been associated with the development of lung function impairment after acute COVID-19 [43, 44]. Our data indicated that a decreased serum CD4/CD8 ratio was independently associated with SLH. Taken together, a decreased CD4/CD8 ratio because of a reduced CD4 + T-cell count and increased CD8 + T-cell count may cause SAD and, eventually, SLH. Further research is warranted to explore the relationship between dysregulated CD4/CD8 immune response, SAD, and SLH development in patients with PASC.

This study has several limitations. First, lung function parameters were not obtained from participants prior to SARS-COV-2 infection. To minimize possible confounders such as SAD and lung function abnormalities, participants with chronic lung disease or lung cancer were excluded. Second, participants were enrolled primarily during the pandemic of the delta variant of COVID-19, and most participants did not receive the vaccine for COVID-19. Antiviral agents were all used in our participants during the acute infection phase (2 months before data collection at visit 1). Due to the small sample size, we were unable to properly group participants with different antiviral agent, which prevented us from conducting an analysis on their effects. It is worth noting that a recently published randomized controlled trial (RCT) did not demonstrate long-term benefits for remdesivir in patients hospitalized due to COVID-19 [45]. The effectiveness of Nirmatrelvir–Ritonavir against long COVID remains inconclusive [46–48]. Molnupiravir treatment reduces the risk of long COVID in high-risk patients but does not decrease the risk of dyspnea or cough [49]. Thus, we could not evaluate the effects of vaccine immunization, anti-viral agent and subsequent immune reactions on the small airways of participants during the study period. Third, the number of patients in each group was relatively small. Therefore, the generalizability of our findings may be limited. Fourth, The Global Lung Function Initiative (GLI) reference equations for static lung volume indicate that the RV/TLC ratio increases with age in individuals over 40 years old [50]. Currently, in Taiwan, we do not have the upper limit of normal (ULN) for RV/TLC ratios, and the GLI reference equations are designed to fit individuals of European ancestry. Using the absolute RV/TLC ratio rather than the ULN may lead to misclassification of patients. Finally, the follow-up period was insufficient to determine when, if at all, the SAD would return to normal and whether managing lung hyperinflation could improve clinical symptoms, dyspnea, and fatigue in patients with PASC. Prospective studies with longer follow-up periods and treatment of SLH are urgently needed.

## Conclusion

In conclusion, SAD measured using IOS and a decreased serum CD4/CD8 ratio correlated with SLH development in patients with PASC. SLH was common among patients with PASC, irrespective of COVID-19 severity, and was independently associated with the long COVID symptoms of dyspnea and fatigue. Additionally, SAD was significantly associated with post-COVID breathlessness. These findings suggest that SLH may persist in some patients even after recovering from acute SARS-CoV-2 infection. Therefore, future studies should focus on exploring the underlying mechanisms of SAD in patients with PASC to develop targeted therapeutic interventions. Furthermore, considering the persistence of SAD in association with SLH, long-term assessments of patients with PASC are warranted to better understand and manage their respiratory health.

### Electronic supplementary material

Below is the link to the electronic supplementary material.

Supplementary Material 1

## Data Availability

We cannot share individual-level data owing to data protection rules.

## Abbreviations

aOR: Adjusted odds ratio
AX: Area under reactance curve between 5 Hz and resonant frequency
BMI: Body mass index
CI: Confidence interval
DLCO: Diffusing capacity of the lung for carbon monoxide
FEV_1_: Forced expiratory volume in the first second
Fres: Resonant frequency
FVC: Forced vital capacity
IOS: Impulse oscillometry
PASC: Post-acute sequelae of COVID-19
RV/TLC: Residual volume to total lung capacity ratio
R5-: R20 Difference between resistance at 5 and 20 Hz
SAD: Small airway dysfunction
SARS-: CoV-2 Severe acute respiratory syndrome coronavirus 2
SGRQ: St George’s Respiratory Questionnaire
SLH: Static lung hyperinflation
X5: Respiratory reactance at 5 Hz
