# Supplementary material for: Long COVID symptoms after 8-month recovery: persistent static lung hyperinflation associated with small airway dysfunction
Source: Respir Res. 2024 May 15;25:209. doi: 10.1186/s12931-024-02830-1 (PMC11097537; doi:10.1186/s12931-024-02830-1)
Supplement: Supplementary file 1 — Supplementary Material 1 [file 12931_2024_2830_MOESM1_ESM.docx]

**Supplementary materials**

**Method**

The equations are as below. The predicted equations for spirometry and body plethysmography are generated from Taiwanese healthy subjects who underwent lung function tests in our department.

FVC (L)

Male adult = 0.05045 x Height(cm) – 0.02253x Age -3.40641

Female adult = 0.04232 x Height(cm) – 0.01531x Age -3.01855

FEV1(L)

Male adult = 0.03419 x Height(cm) – 0.03032x Age -1.11927

Female adult = 0.03466 x Height(cm) – 0.01903x Age -2.12547

TLC (L)

Male adult = 0.05032x Height(cm)-0.00345 x Age -3.40667

Female adult = 0.050320 x Height (cm)- 0.00345x Age -3.40667

IC (L)

Male adult = 0.03566x Height(cm)-0.01106 x Age -2.96417

Female adult = 0.01943 x Height (cm)- 0.00584 x Age -0.94147

FRC (L)

Male adult = 0.05930 x Height(cm)-0.01555 x BW (kg) -6.04269

Female adult = 0.03518 x Height (cm)- 0.01458 x BW (kg) -2.47813

| **Table A. Symptoms of Patients With PASC** | |
| --- | --- |
| Symptom n (%) | PASC  (n=64) |
| Fatigue | 37(57.8) |
| Respiratory symptoms |  |
| Dyspnea | 28(43.8) |
| Cough | 28(43.8) |
| Mucus secretion | 30(46.9) |
| Cardiac symptoms |  |
| Chest tightness | 19(29.7) |
| Palpitation | 16(25.0) |
| Sleep difficulties | 24(37.5) |
| Muscle weakness | 17(26.5) |
| Joint pain | 19(29.7) |
| Myalgia | 17(26.5) |
| Hair loss | 26(40.6) |
| Smell disorder | 7(10.9) |
| Taste disorder | 4(6.2) |
| Decreased appetite | 11(17.1) |
| Vomiting | 5(7.8) |
| Diarrhea | 6(9.3) |
| Headache | 18(28.1) |
| Dizziness | 16(25.0) |
| Brain fog | 32(50) |
| Skin rash | 10(15.6) |
| PASC: post-acute sequelae of COVID-19. | |

| **Table B. Odds Ratios (ORs) (95% CIs) for Persistent SLH in Patients With PASC** | | | | |
| --- | --- | --- | --- | --- |
| Clinical variable | OR (95% CI) | p-value | Adjusted OR  (95% CI) | p-value |
| Age, yrs | 1.26 (1.10-1.44) | **0.001** | 1.33 (1.10-1.61) | **0.03** |
| Sex (female) | 0.26 (0.05-1.33) | 0.106 |  |  |
| BMI | 0.88 (0.74-1.04) | 0.133 |  |  |
| History of smoking | 4.00 (0.38-41.74) | 0.247 |  |  |
| COVID-19 severity(mild) | 0.39 (0.03-5.21) | 0.476 |  |  |
| FVC%pred | 0.94 (0.88-1.00) | **0.036** | 0.91 (0.81-1.02) | 0.089 |
| FEV1%pred | 0.99 (0.93-1.05) | 0.691 |  |  |
| FEV1/FVC % | 1.14 (1.00-1.30) | 0.052 | 1.39 (1.02-1.90) | **0.038** |
| FEF25-75%pred | 1.02 (0.99-1.04) | 0.228 |  |  |
| DLCO% | 0.93 (0.86-1.00) | **0.037** | 0.90 (0.79-1.03) | 0.132 |
| Dyspnea | 14.00 (2.08-94.24) | **0.007** | 628.7 (6.28-62966.85) | **0.006** |
| Fatigue | 6.6 (1.23-35.44) | **0.028** | 121.0 (2.37-6173.33) | **0.0017** |
| R5-R20>0.07 kPa/(L/s) | 4.2 (0.84-21.5) | 0.081 | 113.75 (1.59-8123.96) | **0.03** |
| AX>0.44 kPa/L | 3.86 (0.75-19.84) | 0.106 | 7.38 (0.292-186.21) | 0.225 |
| X5<-0.12 kPa/(L/s) | 0.88 (0.19-4.00) | 0.863 |  |  |
| Fres>14.4 Hz | 3.0 (0.59-15.36) | 0.187 |  |  |
| 6MWD | 1.00 (0.99-1.00) | 0.236 |  |  |
| SGRQ score | 1.02 (0.97-1.07) | 0.385 |  |  |
| CD4+ T cell, cell/uL | 0.08(0.002-3.20) | 0.178 |  |  |
| CD8+ T cell, cell/uL | 3.16 (0.19-53.31) | 0.424 |  |  |
| CD4/CD8 T cell ratio | 0.48 (0.20-1.12) | 0.09 | 0.37 (0.08-1.60) | 0.181 |
| CD19+ B cell, cell/uL | 0.76 (0.60-0.95) | **0.015** | 0.70 (0.50-0.99) | **0.042** |
|  |  |  |  |  |
| We used a multinominal regression model (non-SLH group versus variable SLH group versus persistent SLH group) to predict persistent SLH and adjusted it with age, sex, BMI, smoking history, and COVID-19 severity.  % pred.: % of predicted value, 6MWD: 6-min walking distance, AX: area under reactance curve between 5 Hz and resonant frequency, BMI: body mass index, DLCO: diffusing capacity of the lung for carbon monoxide, FEV1: forced expiratory volume in 1 s; FEF25-75%: Forced expiratory flow at 25% to 75% of FVC; Fres: resonant frequency, FVC: forced vital capacity, PASC: post-acute sequelae of COVID-19, R5-R20: difference between resistance at 5 and 20 Hz, SGRQ: St George’s Respiratory Questionnaire, SLH: static lung hyperinflation. X5: reactance in 5 Hz. | | | | |

| **Table C1 Odds Ratios (ORs) (95% CIs) for Clinical Variables Associated With Presence of Dyspnea in Patients With PASC at Visit 2** | | | | |
| --- | --- | --- | --- | --- |
| Clinical variables | Univariable | | Multivariable | |
|  | OR (95% CI) | p-value | OR (95% CI) | p-value |
| Age, yrs | 1.03 (0.99-1.08) | 0.176 | 0.97 (0.87-1.07) | 0.535 |
| Sex | 3.27 (1.02-10.46) | 0.046 | 2.98 (0.74-12.04) | 0.126 |
| BMI | 1.03 (0.93-1.15) | 0.561 |  |  |
| History of smoking | 0.59 (0.25-1.37) | 0.218 |  |  |
| COVID-19 severity | 0.72 (0.34-1.51) | 0.386 |  |  |
| FVC%pred | 1.02 (0.97-1.07) | 0.432 |  |  |
| FEV1%pred | 1.05 (0.99-1.11) | 0.101 | 1.05 (0.98-1.11) | 0.162 |
| FEV1/FVC % | 1.04 (0.94-1.15) | 0.488 |  |  |
| FEF25-75%pred | 1.01 (0.99-1.03) | 0.349 |  |  |
| DLCO% | 0.98 (0.94-1.02) | 0.356 |  |  |
| Presence of SLH | 7.80  (2.24-27.16) | **0.001** | 9.73 (1.87-50.65) | **0.007** |
| R5-R20>0.07 kPa/(L/s) | 1.93 (0.61-6.09) | 0.263 |  |  |
| AX>0.44 kPa/L | 2.47 (0.77-7.91) | 0.128 | 1.22 (0.31-4.83) | 0.78 |
| X5<-0.12 kPa/(L/s) | 1.03 (0.34-3.10) | 0.959 |  |  |
| Fres>14.4 Hz | 2.25 (0.73-6.91) | 0.156 |  |  |
| 6MWD | 0.99 (0.99-1.00) | 0.026 | 0.99 (0.98-1.00) | 0.086 |
| SGRQ score | 1.10 (1.03-1.16) | **0.003** | 1.08 (1.02-1.15) | **0.001** |
| Bronchodilator usage | 1.17 (0.32-4.20) | 0.814 |  |  |

% pred.: % of predicted value, 6MWD: 6-min walking distance, AX: area under reactance curve between 5 Hz and resonant frequency, BMI: body mass index, DLCO: diffusing capacity of the lung for carbon monoxide, FEV1: forced expiratory volume in 1 s; FEF25-75%: Forced expiratory flow at 25% to 75% of FVC; Fres: resonant frequency, FVC: forced vital capacity, PASC: post-acute sequelae of COVID-19, R5-R20: difference between resistance at 5 and 20 Hz, SGRQ: St George’s Respiratory Questionnaire, SLH: static lung hyperinflation. X5: reactance in 5 Hz.

| **Table C2 Odds Ratios (ORs) (95% CIs) for Clinical Variables Associated With Presence of Fatigue in Patients With PASC at Visit 2** | | | | |
| --- | --- | --- | --- | --- |
| Clinical variables | Univariable | | Multivariable | |
|  | OR (95% CI) | p-value | OR (95% CI) | p-value |
| Age, yrs | 1.04 (1.00-1.09) | 0.064 | 1.05 (1.00-1.11) | **0.038** |
| Sex | 1.32 (0.46-3.79) | 0.611 |  |  |
| BMI | 1.02 (0.92-1.13) | 0.731 |  |  |
| History of smoking | 1.35 (0.66-2.76) | 0.41 |  |  |
| COVID-19 severity | 0.92 (0.47-1.81) | 0.805 |  |  |
| FVC%pred | 1.02 (0.98-1.07) | 0.335 |  |  |
| FEV1%pred | 1.05 (1.00-1.11) | 0.065 | 1.04 (0.98-1.10) | 0.197 |
| FEV1/FVC % | 0.98 (0.90-1.09) | 0.795 |  |  |
| FEF25-75%pred | 1.01 (0.99-1.03) | 0.383 |  |  |
| DLCO% | 0.38 (0.17-0.86) | **0.019** | 0.98 (0.94-1.03) | 0.430 |
| Presence of SLH | 7.28 (2.14-24.77) | **0.002** | 11.59 (2.23-60.41) | **0.004** |
| R5-R20>0.07 kPa/(L/s) | 1.05 (0.35-3.11) | 0.933 |  |  |
| AX>0.44 kPa/L | 1.58 (0.54-4.65) | 0.404 |  |  |
| X5<-0.12 kPa/(L/s) | 1.87 (0.64-5.51) | 0.256 |  |  |
| Fres>14.4 Hz | 1.49 (0.51-4.37) | 0.465 |  |  |
| 6MWD | 1.00 (0.99-1.00) | 0.253 |  |  |
| SGRQ score | 1.07 (1.01-1.14) | **0.021** | 1.07 (1.00-1.13) | **0.037** |
| Bronchodilator usage | 1.39 (0.39-4.94) | 0.610 |  |  |

% pred.: % of predicted value, 6MWD: 6-min walking distance, AX: area under reactance curve between 5 Hz and resonant frequency, BMI: body mass index, DLCO: diffusing capacity of the lung for carbon monoxide, FEV1: forced expiratory volume in 1 s; FEF25-75%: Forced expiratory flow at 25% to 75% of FVC; Fres: resonant frequency, FVC: forced vital capacity, PASC: post-acute sequelae of COVID-19, R5-R20: difference between resistance at 5 and 20 Hz, SGRQ: St George’s Respiratory Questionnaire, SLH: static lung hyperinflation. X5: reactance in 5 Hz.

| **Table C3 Odds Ratios (ORs) (95% CIs) for Clinical Variables Associated With Presence of Dyspnea in Patients With PASC at Visit 3** | | | | |
| --- | --- | --- | --- | --- |
| Clinical variables | Univariable | | Multivariable | |
|  | OR (95% CI) | p-value | OR (95% CI) | p-value |
| Age, yrs | 1.07 (1.01-1.13) | 0.026 | 1.15 (1.05-1.27) | **0.004** |
| Sex | 3.95 (1.05-14.85) | 0.042 | 9.35 (0.73-119.54) | 0.086 |
| BMI | 1.08 (0.96-1.20) | 0.192 |  |  |
| History of smoking | 0.64 (0.27-1.51) | 0.303 |  |  |
| COVID-19 severity | 0.44 (0.17-1.16) | 0.096 | 0.20 (0.05-0.84) | 0.028 |
| FVC%pred | 0.94 (0.89-1.00) | 0.036 | 0.90 (0.82-0.99) | 0.028 |
| FEV1%pred | 0.96 (0.91-1.02) | 0.183 |  |  |
| FEV1/FVC % | 1.04 (0.93-1.17) | 0.496 |  |  |
| FEF25-75%pred | 1.01 (0.98-1.03) | 0.638 |  |  |
| DLCO% | 0.97 (0.92-1.02) | 0.261 |  |  |
| Presence of SLH | 11.52  (2.78-47.67) | **0.001** | 12.36 (1.34-114.32) | **0.027** |
| R5-R20>0.07 kPa/(L/s) | 7.64 (1.78-32.72) | 0.006 | 12.84 (1.31-125.71) | **0.028** |
| AX>0.44 kPa/L | 7.64 (1.78-32.72) | 0.006 | 7.60 (1.14-50.48) | **0.036** |
| X5<-0.12 kPa/(L/s) | 5.60 (1.52-20.61) | 0.01 | 1.83 (0.32-10.53) | 0.497 |
| Fres>14.4 Hz | 1.83 (0.54-6.24) | 0.332 |  |  |
| 6MWD | 1.00 (0.99-1.00) | 0.151 |  |  |
| SGRQ score | 1.18 (1.06-1.32) | **0.002** | 1.18 (1.02-1.37) | **0.03** |
| Bronchodilator usage | 1.23 (0.29-5.16) | 0.777 |  |  |

% pred.: % of predicted value, 6MWD: 6-min walking distance, AX: area under reactance curve between 5 Hz and resonant frequency, BMI: body mass index, DLCO: diffusing capacity of the lung for carbon monoxide, FEV1: forced expiratory volume in 1 s; FEF25-75%: Forced expiratory flow at 25% to 75% of FVC; Fres: resonant frequency, FVC: forced vital capacity, PASC: post-acute sequelae of COVID-19, R5-R20: difference between resistance at 5 and 20 Hz, SGRQ: St George’s Respiratory Questionnaire, SLH: static lung hyperinflation. X5: reactance in 5 Hz.

| **Table C4 Odds Ratios (ORs) (95% CIs) for Clinical Variables Associated With Presence of Fatigue in Patients With PASC at Visit 3** | | | | |
| --- | --- | --- | --- | --- |
| Clinical variables | Univariable | | Multivariable | |
|  | OR (95% CI) | p-value | OR (95% CI) | p-value |
| Age, yrs | 1.04 (0.99-1.09) | 0.173 | 1.06 (1.00-1.13) | **0.047** |
| Sex | 2.17 (0.64-7.33) | 0.214 |  |  |
| BMI | 1.08 (0.97-1.21) | 0.179 |  |  |
| History of smoking | 1.05 (0.50-2.18) | 0.904 |  |  |
| COVID-19 severity | 1.08 (0.49-2.38) | 0.844 |  |  |
| FVC%pred | 0.95 (0.90-1.00) | 0.061 | 0.92 (0.86-0.99) | **0.023** |
| FEV1%pred | 0.99 (0.94-1.05) | 0.729 |  |  |
| FEV1/FVC % | 1.19 (1.03-1.37) | **0.02** | 1.30 (1.05-1.60) | **0.015** |
| FEF25-75%pred | 1.03 (1.00-1.07) | 0.031 | 1.05 (1.01-1.09) | **0.025** |
| DLCO% | 0.97 (0.92-1.01) | 0.160 |  |  |
| Presence of SLH | 5.76  (1.56-21.33) | **0.009** | 5.94 (1.01-35.07) | **0.049** |
| R5-R20>0.07 kPa/(L/s) | 1.28 (0.39-4.17) | 0.686 |  |  |
| AX>0.44 kPa/L | 1.85 (0.56-6.13) | 0.316 |  |  |
| X5<-0.12 kPa/(L/s) | 3.62 (1.06-12.31) | **0.039** | 1.83 (0.42-8.03) | 0.422 |
| Fres>14.4 Hz | 0.83 (0.26-2.69) | 0.760 |  |  |
| 6MWD | 1.00 (0.99-1.00) | 0.349 |  |  |
| SGRQ score | 1.11 (1.02-1.22) | **0.019** | 1.08 (0.98-1.19) | 0.134 |
| Bronchodilator usage | 1.64 (0.40-6.71) | 0.489 |  |  |

% pred.: % of predicted value, 6MWD: 6-min walking distance, AX: area under reactance curve between 5 Hz and resonant frequency, BMI: body mass index, DLCO: diffusing capacity of the lung for carbon monoxide, FEV1: forced expiratory volume in 1 s; FEF25-75%: Forced expiratory flow at 25% to 75% of FVC; Fres: resonant frequency, FVC: forced vital capacity, PASC: post-acute sequelae of COVID-19, R5-R20: difference between resistance at 5 and 20 Hz, SGRQ: St George’s Respiratory Questionnaire, SLH: static lung hyperinflation. X5: reactance in 5 Hz.

**Table D. Comparison pulmonary function and symptoms of PASC patients with or without usage of bronchodilators between visit 1 and 3**

|  | Bronchodilators usage | | | | No bronchodilators usage | | |
| --- | --- | --- | --- | --- | --- | --- | --- |
| Characteristic | V1 | | V3 | p-value | V1 | V3 | p-value |
| Numbers | 14 | | 10 |  | 50 | 37 |  |
| Presence of SLH (%) | 8 (57.1) | | 5 (50.0) | 0.50 | 26 (52.0) | 12 (32.4) | **0.012** |
| Pulmonary Function |  | |  |  |  |  |  |
| FVC, % pred. | 84.3 ± 11.9 | | 89.6 ± 10.8 | 0.059 | 90.6 ± 14.7 | 97.6 ± 10.5 | **<0.001** |
| FEV1, % pred | 89.1 ± 6.6 | | 94.9± 6.0 | **0.041** | 97.9 ± 13.3 | 103.5± 10.6 | **0.002** |
| FEV1/FVC, % | 84.7 ± 6.1 | | 83.3 ± 5.0 | **0.015** | 85.2 ± 6.7 | 83.4 ± 5.1 | **0.008** |
| FEF25%-75%, % pred | 86.1 ± 25.1 | | 87.7 ± 19.1 | 0.594 | 95.4 ± 29.8 | 91.6 ± 25.2 | 0.115 |
| TLC, % pred. | 90.3 ± 12.0 | | 98.1 ± 10.3 | **0.005** | 98.4 ± 14.3 | 103.5 ± 10.7 | 0.078 |
| RV/TLC, % | 39.6 ± 5.9 | | 40.4 ± 7.5 | 0.574 | 40.1 ± 6.7 | 38.5 ± 7.5 | **0.042** |
| DLCO, % pred. | 59.3 ± 14.8 | | 70.2 ± 11.9 | 0.086 | 63.9 ± 13.4 | 67.7 ± 13.4 | **0.044** |
| R5-R20 [kPa/(L/s)] | 0.09 (0.05-0.12) | | 0.08 (0.03-0.09) | 0.326 | 0.07 (0.04-0.12) | 0.08 (0.05-0.11) | 0.950 |
| R5-R20 > 0.07(%) | | 8 (57.1) | 5 (50.0) | 1.00 | 20 (40.0) | 20 (54.1) | 0.092 |
| AX (kPa/L) | 0.65 (0.25-0.77) | | 0.43 (0.13-0.83) | 0.445 | 0.46 (0.28-0.96) | 0.49 (0.21-0.73) | 0.831 |
| AX > 0.44 (%) | 9 (64.3) | | 5 (50.0) | 1.00 | 27 (0.54) | 20 (54.1) | 1.00 |
| X5 [kPa/(L/s)] | -0.13 (-0.17- -0.06) | | -0.13 (-0.2- -0.07) | 0.759 | -0.12 (-0.18- -0.09) | -0.12 (-0.14- -0.09) | 0.576 |
| X5 < -0.12(%) | 8 (57.1) | | 5 (50.0) | 1.00 | 27 (0.54) | 16 (43.2) | 0.344 |
| Fres (Hz) | 15.7 (12.9-17.1) | | 13.6 (8.8-15.0) | 0.445 | 14.7 (11.9-17.2) | 14.6 (12.7-16.0) | 0.815 |
| Fres > 14.14(%) | 10 (71.4) | | 4 (40.0) | 0.625 | 27 (0.54) | 22 (59.4) | 0.791 |
| SAD (%) | 10 (71.4) | | 6 (60.0) | 1.00 | 32 (64.0) | 27 (73.0) | 0.453 |
| Symptoms |  | |  |  |  |  |  |
| Fatigue (%) | 10(57.8) | | 5 (50.0) | 0.50 | 27 (54.0) | 14 (37.8) | **0.049** |
| Dyspnea (%) | 7(43.8) | | 4(40.0) | 1.00 | 21 (42.0) | 13 (35.1) | 0.607 |
| Cough (%) | 7 (43.8) | | 0 (0) | 0.25 | 21 (42.0) | 10 (27.0) | 0.227 |
| Mucus secretion (%) | 6 (46.9) | | 2 (20.0) | 1.00 | 24 (48.0) | 17 (45.9) | 1.000 |

% pred.: % of predicted value, AX: area under reactance curve between 5 Hz and resonant frequency, DLCO: diffusing capacity of the lung for carbon monoxide, FEV1: forced expiratory volume in 1 s; FEF25%–75%: Forced expiratory flow at 25% to 75% of FVC, Fres: resonant frequency, FVC: forced vital capacity, PASC: post-acute sequelae of COVID-19, R5-R20: difference between resistance at 5 and 20 Hz, SAD: small airway dysfunction, SLH: static lung hyperinflation, V1: visit one, V2: visit two. X5: reactance in 5 Hz. Bold text indicates that the p value is significant.

The cutoffs for SAD were the difference between resistance at 5 and 20 Hz (R5-R20) greater than 0.07 kPa/(L/s), the area under the reactance curve between 5 Hz and the resonant frequency (AX) greater than 0.44 kPa/L, X5 less than - 0.12 kPa/(L/s), or resonant frequency (Fres) greater than 14.14 Hz
